# Supplementary material for: Maximizing Cumulative Trypsin Activity with Calcium at Elevated Temperature for Enhanced Bottom-Up Proteome Analysis
Source: Biology (Basel). 2022 Oct 1;11(10):1444. doi: 10.3390/biology11101444 (PMC9598648; doi:10.3390/biology11101444)
Supplement: Supplementary file 1 [file biology-11-01444-s001.zip › biology-1907174-supplementary.pdf]

Supplementary Material

Maximizing cumulative trypsin activity with calcium at elevated temperature for enhanced bottom-up proteome analysis.

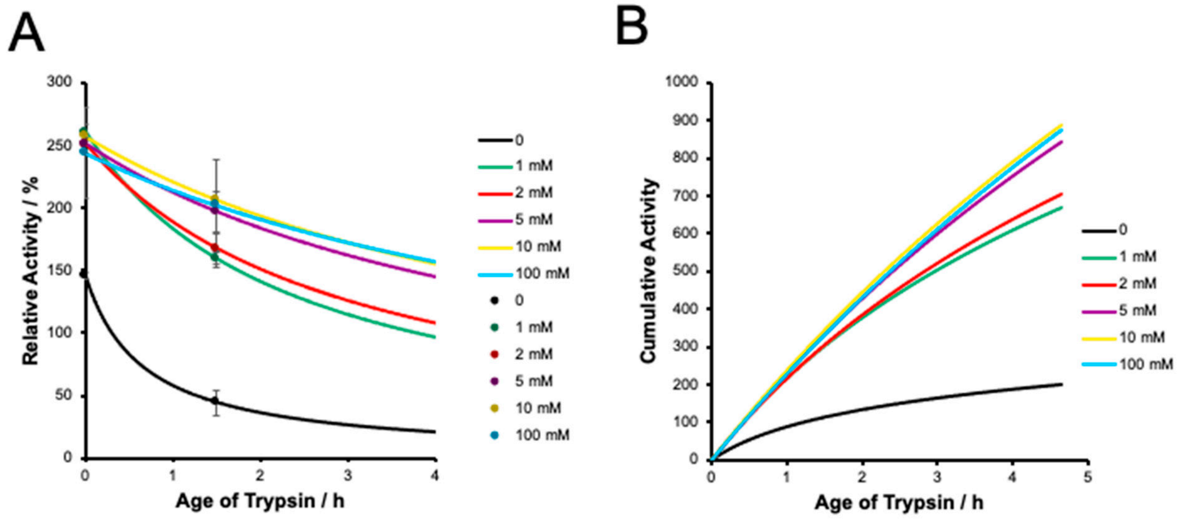

Supplementary Figure S1. (**A**) Second-order kinetics models of trypsin de-activation at 47 °C with 0 – 100 mM added calcium chloride. (**B**) Estimated cumulative activity based on integrated second-order kinetics models.

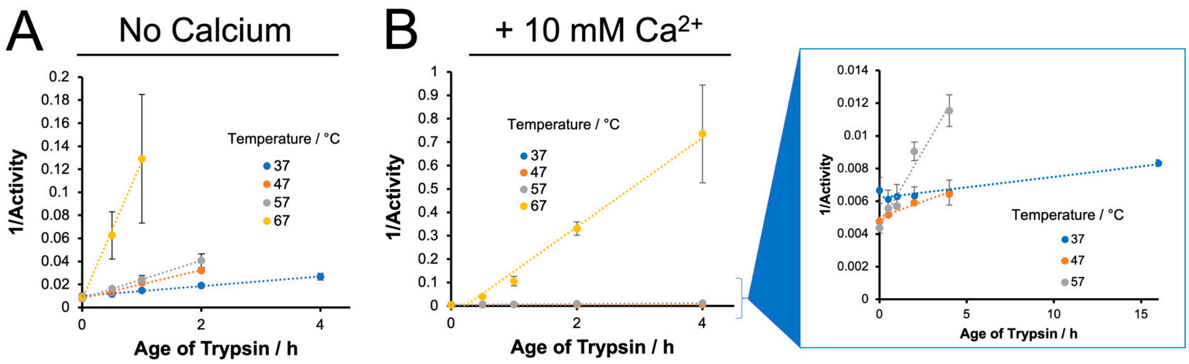

Supplementary Figure S2. Second-order kinetics models of trypsin de-activation at 37-67 °C (**A**) with no added calcium ions, and (**B**) with 10 mM added calcium chloride.

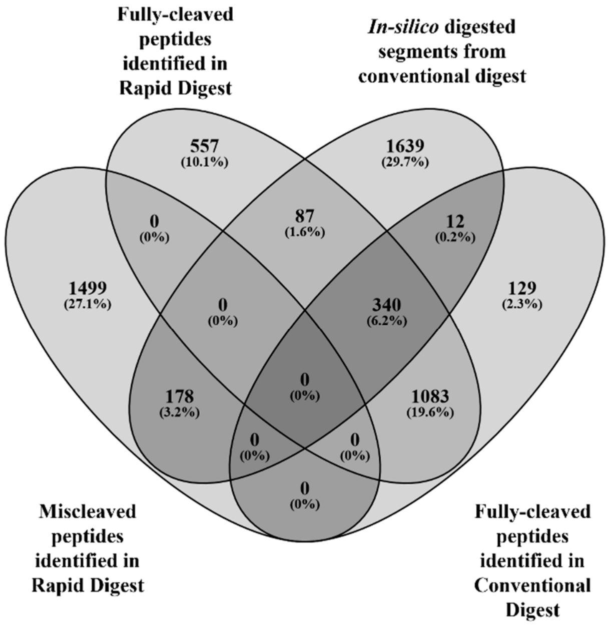

Supplementary Figure S3. Venn diagram comparing *in-silico* digestion of miscleaved peptides identified in the conventional digest with peptides identified in both the rapid and conventional digests.

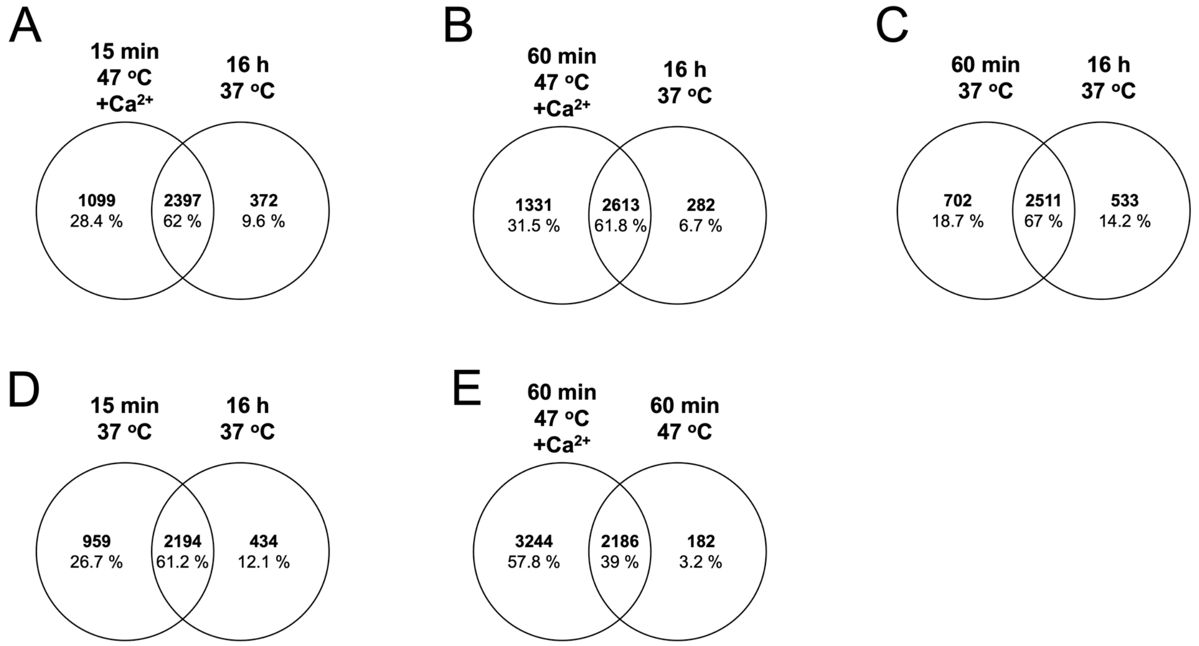

Supplementary Figure S4. Venn diagrams of bottom-up peptide identifications compared across the "light" and "heavy" label within each LC-MS/MS injection.

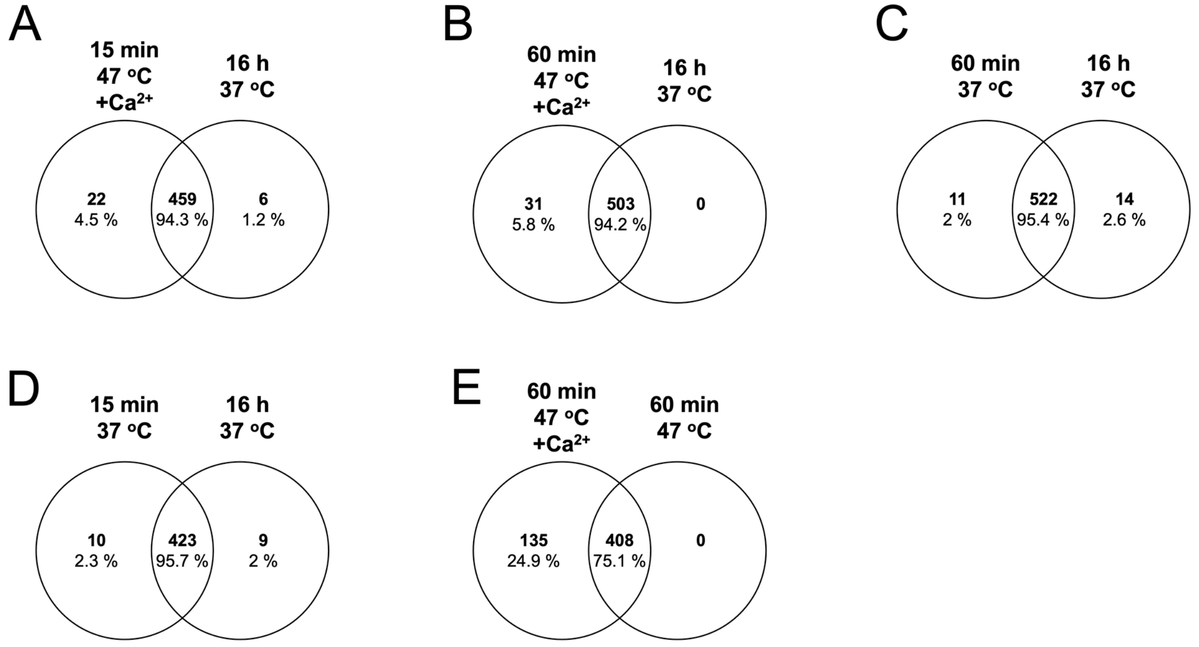

Supplementary Figure S5. Venn diagrams of bottom-up protein identifications compared across the "light" and "heavy" label within each LC-MS/MS injection.

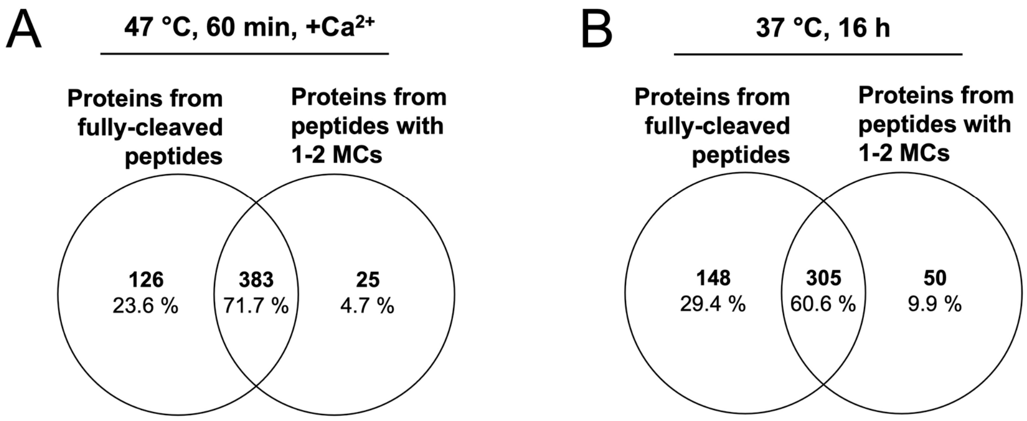

Supplementary Figure S6. Venn diagrams of bottom-up protein identifications contributed by fully-cleaved peptides vs. those with 1-2 miscleavages in (**A**) the enhanced rapid digest and (**B**) the conventional/control digest.

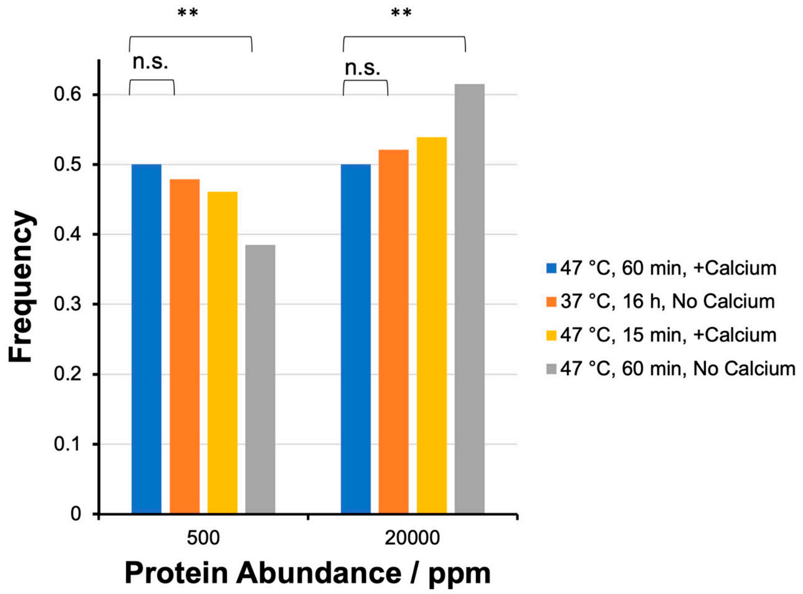

Supplementary Figure S7. Histogram of the frequency of identifying low vs. high abundance proteins in the experimental digestion confitions, showing the greatest frequency of low-abundance proteins in the optimized rapid digest (60 min at 47 °C with 10 mM added calcium ions).
